# Supplementary material for: Investigation and public health response to a COVID-19 outbreak in a rural resort community—Blaine County, Idaho, 2020
Source: PLoS One. 2021 Apr 21;16(4):e0250322. doi: 10.1371/journal.pone.0250322 (PMC8059800; doi:10.1371/journal.pone.0250322)
Supplement: S3 Table — (PDF) [file pone.0250322.s003.pdf]

S3 Table. Distribution of confirmed COVID-19 cases (n = 402) and census block group characteristics for Blaine County, Idaho. Census block group data are 2014-2018 American Community Survey 5-year estimates. Methods are described below.

		Census Block Group Characteristics						
					Median household income (USD)			
**Census block group**	**COVID-19 cases (%)**	**Population**	**Hispanic or Latino origin**	**Persons in renter-occupied housing**	**under $25K**	**$25K to $75K**	**$75K to $150K**	**over $150K**
Block Group 1, Census Tract 9601	3 (0.7)	1304	12.9%	40.8%	25.5%	39.3%	25.2%	10.0%
Block Group 2, Census Tract 9601	11 (2.7)	1421	15.6%	37.2%	22.3%	52.7%	18.3%	6.8%
Block Group 3, Census Tract 9601	23 (5.7)	1478	4.1%	13.7%	18.0%	42.8%	31.5%	7.6%
Block Group 4, Census Tract 9601	14 (3.5)	571	29.8%	37.2%	29.5%	41.5%	29.0%	0.0%
Block Group 1, Census Tract 9602	37 (9.2)	1313	20.0%	54.1%	46.4%	39.0%	8.3%	6.4%
Block Group 2, Census Tract 9602	44 (10.9)	2667	6.5%	24.3%	15.3%	28.8%	32.8%	23.1%
Block Group 3, Census Tract 9602	70 (17.4)	5052	47.1%	49.2%	19.3%	49.5%	25.9%	5.4%
Block Group 4, Census Tract 9602	15 (3.7)	1346	41.8%	13.5%	6.5%	56.4%	32.4%	4.7%
Block Group 1, Census Tract 9603	48 (11.9)	1043	4.6%	11.1%	15.4%	37.6%	33.4%	13.6%
Block Group 2, Census Tract 9603	19 (4.7)	878	14.4%	33.0%	27.9%	35.8%	29.3%	7.0%
Block Group 3, Census Tract 9603	54 (13.4)	1800	10.9%	49.0%	13.0%	50.2%	30.8%	6.0%
Block Group 1, Census Tract 9605	32 (8.0)	1835	21.2%	25.8%	27.4%	28.8%	32.7%	11.1%
Block Group 2, Census Tract 9605	32 (8.0)	1286	3.3%	21.7%	20.1%	45.2%	23.5%	11.2%

Census block group analysis methods

Patient addresses were geocoded using Texas A&M Geocoding Services (http://geoservices.tamu.edu/Services/Geocode/) to obtain census block group identifiers. Block groups are statistical divisions of census tracts that typically cover contiguous areas and contain between 600 and 3,000 people (https://www.census.gov/programs-surveys/geography/about/glossary.html). For each block group, median household income, the proportion of Hispanic or Latino residents, and renter-occupied housing units was obtained using 2014–2018 American Community Survey 5-year estimates downloaded from https://data.census.gov/cedsci/.
