# Supplementary material for: Investigation and public health response to a COVID-19 outbreak in a rural resort community—Blaine County, Idaho, 2020
Source: PLoS One. 2021 Apr 21;16(4):e0250322. doi: 10.1371/journal.pone.0250322 (PMC8059800; doi:10.1371/journal.pone.0250322)
Supplement: S4 Table — (PDF) [file pone.0250322.s004.pdf]

S4 Table. Univariable and multivariable analysis of COVID-19 patient characteristics associated with hospitalization.

Variable	Hospitalized n/N (%)	Unadjusted odds ratio (95%CI)	P value	Adjusted odds ratio (95% CI)	P value
**Age group**					
<18 years	0/5 (0)	n/a*		n/a*	
18 to 44 years	5/169 (3.0)	ref		ref	
45 to 64 years	14/172 (8.1)	2.9 (1.0–8.3)	0.045	4.0 (1.0–15.5)	0.043
≥ 65 years	33/105 (31.4)	15.0 (5.6–40.1)	<0.001	23.0 (5.6–94.4)	<0.001
**Sex**					
Female	23/239 (9.6)	ref		ref	
Male	29/212 (13.7)	1.5 (0.8–2.7)	0.181	1.8 (0.8–3.8)	0.137
**Race/Ethnicity**					
White	34/332 (10.2)	ref		ref	
Hispanic or Latino	10/73 (13.7)	1.4 (0.7–3.0)	0.392	3.6 (1.2–10.5)	0.020
Other	0/9 (0)	n/a*		n/a*	
**Healthcare worker**					
No	48/394 (12.2)	ref		ref	
Yes	4/56 (7.1)	0.6 (0.2–1.6)	0.276	1.2 (0.3–4.3)	0.800
**Number of symptoms**					
0 to 4	20/255 (7.8)	ref		ref	
5 or more	32/187 (17.1)	2.4 (1.3–4.4)	0.004	3.1 (1.5–6.4)	0.002

*Excluded from analysis because of small sample size.
