# Supplementary material for: Investigation and public health response to a COVID-19 outbreak in a rural resort community—Blaine County, Idaho, 2020
Source: PLoS One. 2021 Apr 21;16(4):e0250322. doi: 10.1371/journal.pone.0250322 (PMC8059800; doi:10.1371/journal.pone.0250322)
Supplement: S1 Fig — (PDF) [file pone.0250322.s005.pdf]

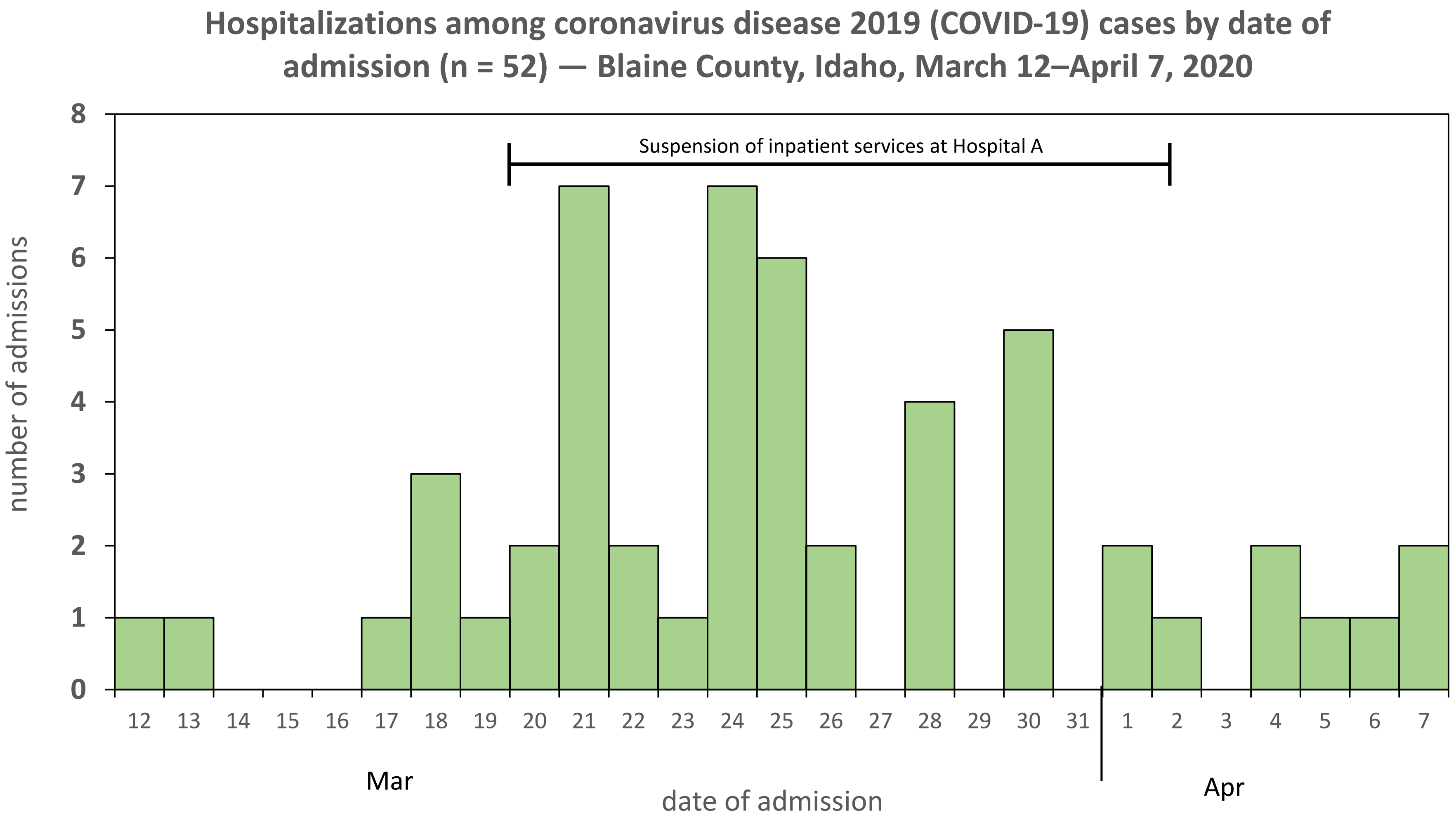

S1 Fig. Hospitalization of Blaine County residents for COVID-19 by date of admission. Admission dates from cases reported from March 13–April 10 are included on the graph.
