# Supplementary material for: Investigation and public health response to a COVID-19 outbreak in a rural resort community—Blaine County, Idaho, 2020
Source: PLoS One. 2021 Apr 21;16(4):e0250322. doi: 10.1371/journal.pone.0250322 (PMC8059800; doi:10.1371/journal.pone.0250322)
Supplement: S2 Fig — (PDF) [file pone.0250322.s006.pdf]

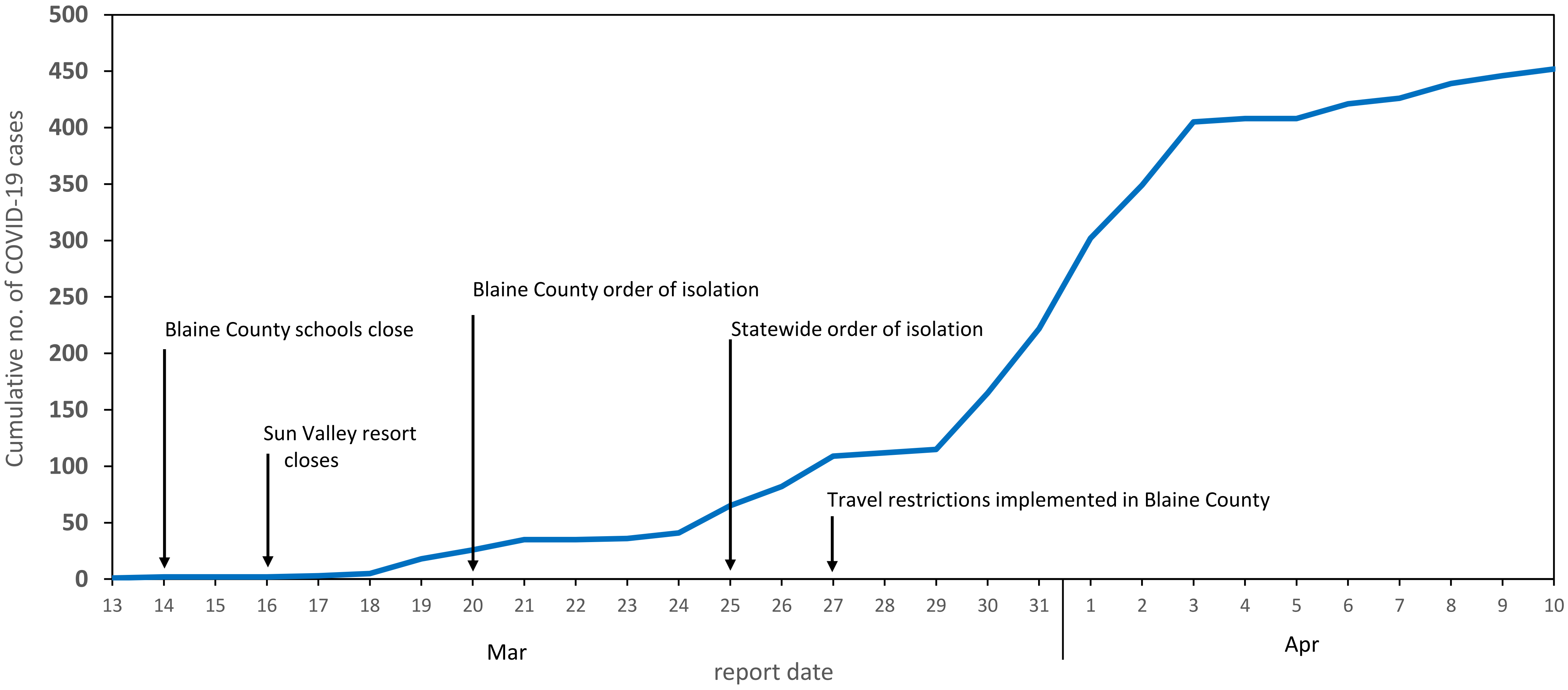

S2 Fig. Timeline of the COVID-19 outbreak in Blaine County, Idaho depicting cumulative number of confirmed COVID-19 cases by date of report and implementation dates for key community mitigation measures. Cases reported during March 13–April 10, 2020 are included in the figure.
